# Distribution and Neurochemistry of the Porcine Ileocaecal Valve Projecting Sensory Neurons in the Dorsal Root Ganglia and the Influence of Lipopolysaccharide from Different Serotypes of *Salmonella* spp. on the Chemical Coding of DRG Neurons in the Cell Cultures

**DOI:** 10.3390/ijms19092551

**Published:** 2018-08-28

**Authors:** Anita Mikołajczyk, Anna Kozłowska, Sławomir Gonkowski

**Affiliations:** 1 Department of Public Health, Faculty of Health Sciences, Collegium Medicum, University of Warmia and Mazury in Olsztyn, Warszawska 30 Str., 10-082 Olsztyn, Poland; 2 Department of Human Physiology, School of Medicine, Collegium Medicum, University of Warmia and Mazury in Olsztyn, Warszawska 30 Str., 10-082 Olsztyn, Poland; kozlowska.anna@uwm.edu.pl; 3 Department of Clinical Physiology, Faculty of Veterinary Medicine, University of Warmia and Mazury in Olsztyn, Oczapowskiego 13 Str., 10-718 Olsztyn, Poland; slawekg@uwm.edu.pl

**Keywords:** ileocecal valve (ICV), LPS from *S.* Enteritidis, LPS from *S.* Minnesota, LPS from *S.* Typhimurium, neuropeptides of DRG

## Abstract

The ileocecal valve (ICV)—a sphincter muscle between small and large intestine—plays important roles in the physiology of the gastrointestinal (GI) tract, but many aspects connected with the innervation of the ICV remain unknown. Thus, the aim of this study was to investigate the localization and neurochemical characterization of neurons located in the dorsal root ganglia and supplying the ICV of the domestic pig. The results have shown that such neurons mainly located in the dorsal root ganglia (DRG) of thoracic and lumbar neuromers show the presence of substance P (SP), calcitonin gene-related peptide (CGRP), and galanin (GAL). The second part of the experiment consisted of a study on the influence of a low dose of lipopolysaccharide (LPS) from *Salmonella* serotypes Enteritidis Minnesota and Typhimurium on DRG neurons. It has been shown that the LPS of these serotypes in studied doses does not change the number of DRG neurons in the cell cultures, but influences the immunoreactivity to SP and GAL. The observed changes in neurochemical characterization depend on the bacterial serotype. The results show that DRG neurons take part in the innervation of the ICV and may change their neurochemical characterization under the impact of LPS, which is probably connected with direct actions of this substance on the nervous tissue and/or its pro-inflammatory activity.

## 1. Introduction

The innervation of the gastrointestinal (GI) tract consists of two parts: the intestinal nervous system (ENS) and extrinsic innervation [1,2,3,4]. The ENS, located in the wall of the oesophagus, stomach, and intestine, is built of millions of neuronal cells divided into ganglionated plexuses, whose quantity depends on animal species [5]. Enteric neurons are very diverse in their morphology, physiology, and neurochemical characteristics and regulate the majority of the functions of the GI tract [1,5]. Due to the significant degree of the independence from the brain, the ENS is called the “second” or “intestinal” brain [6]. However, despite a high degree of autonomy, the ENS is, to some extent, controlled by the central nervous system by the extrinsic innervation of the GI tract. This innervation consists of three fundamental parts, including parasympathetic efferent innervation, sympathetic efferent innervation and afferent innervation [7,8]. The precise localization of neurons participating in the extrinsic innervation of the stomach and intestine clearly depends on innervated segment of the GI tract. In the case of the parasympathetic nervous system, the major part of the GI tract (from oesophagus to transverse colon) is innervated by fibres which are branches of the vagal nerve [7]. Only caudal fragments of the GI tract (descending colon, rectum and anus) are supplied by nerves originating from the parasympathetic nuclei within the intermediolateral column of the sacral spinal cord [9]. In turn, sympathetic neurons innervating the GI tract may be located in the sympathetic chain ganglia and prevertebral ganglia, including the celiac, superior and inferior mesenteric, or pelvic ganglia [2,3,7]. Apart from sympathetic and parasympathetic extrinsic innervation, the GI tract is also supplied by afferent nerves which conduct sensory and pain stimuli from the stomach and intestine to the central nervous system. These nerves are the processes of neuronal cells situated in the nodose ganglia of the vagal nerve or dorsal root ganglia [8]. Previous studies have shown that sensory neurons supplying the gut may contain a wide range of neuronal active substances [8,10]. Among them, the most important factors in sensory and pain stimuli conduction seem to be substance P (SP) and calcitonin gene-related peptide (CGRP). Moreover, it is known that sensory neuronal cells innervating the GI tract may undergo neurochemical changes during pathological processes taking part in the stomach and intestine [8,9,10], but knowledge concerning these aspects is extremely limited.

The ileocecal valve (ICV), a sphincter muscle between small and large intestine, prevents the reflux of colonic content to the ileum and serves as a barrier to the entering of colonic microbial flora to the small intestine [11,12,13]. It should be underlined that previous studies on the innervation of the ICV are few [12] and the sensory neurons innervating this part of the GI tract have not been studied at all. On the other hand, it is known that the organization of the ICV innervation is specific [12,14] and disturbances within it may play important roles in disorders of this part of the GI tract, including constipation, feeling bloated, and/or diarrhoea [12,15]. The knowledge of why the ICV can become impaired, which can lead to many pathological processes such as small intestine bacterial overgrowth (SIBO), is still unclear. This assumes that it is rather the composition of the bacterial species that is more crucial than the number of bacteria. Maybe interaction of some bacteria or some bacterial toxins with the nervous system can also play a role in ICV dysfunction. Due to the above, the study of the interaction between sensory neurons supplying ICV and lipopolysaccharide (LPS) from *Salmonella* spp. seems very interesting.

It should be pointed out that the ileum and cecum were the main sites of *Salmonella* growth in a latent carrier mouse. Thus, parts of the intestine can be strategic places for *Salmonella* proliferation in animals without showing any clinical symptoms of disease during latent infection [16]. Additionally, LPS can be derived from the dead *Salmonella* existing in this part of the intestine. It is known that LPS is present within the cellular membrane of all Gram-negative bacteria and demonstrates negative activity on living organisms [17]. Namely, this substance damages various internal organs, which is connected with the release of free radicals. Moreover, LPS acts on the immunological system, causing fever and septic shock [18]. Previous studies have also shown that LPS may affect the nervous system and is involved in, or connected with, neurodegenerative diseases. However, apart from the fact that LPS may change the expression of the neuronal factor in the internal organs [19,20,21], knowledge concerning changes in neurochemical characterization of neuronal cells under the influence of this substance is lacking. Moreover, it is known that LPS is not a homogeneous substance [22,23,24,25,26]. Additionally, LPS from different species are characterized by various activities [23,27,28] but, until now, the studies on differences in LPS activity derived from various serotypes connected with the influence of this substance on neuronal neurochemical characterization have not yet been studied.

It should be also underlined that the selection of the domestic pig as an experimental animal during this investigation was not accidental. It is relatively well known that the domestic pig is very good animal model of processes occurring in the human organism, due to well confirmed biochemical, neurochemical, and physiological similarities between these species [29]. Thus, the results obtained during this study may reflect the mechanisms connected with the influence of LPS on the human nervous system. The aim of these studies was to investigate the distribution and neurochemistry of the DRG sensory neurons supplying the ICV under physiological conditions Moreover, the influence of a low dose of LPS from various serotypes of *Salmonella* on the neurochemical characterization of neurons located in DRG during the cell cultures has been also studied.

## 2. Results

**Figure 1:**
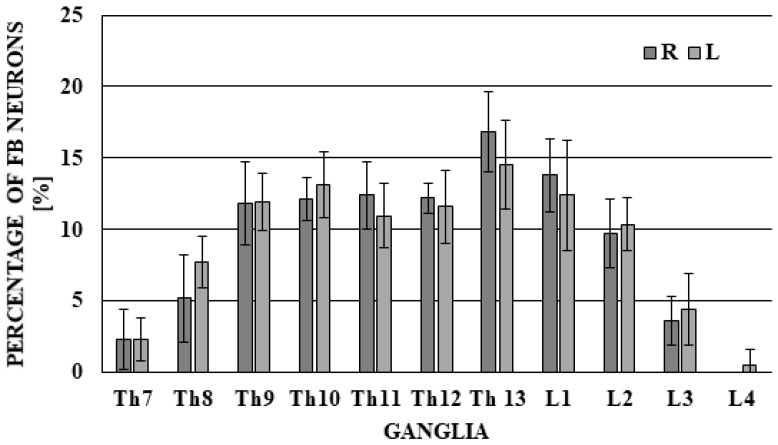
The distribution of fast blue (FB)-labelled neurons supplying the porcine ileocecal valve located in dorsal root ganglia (DRG). The total number of FB-positive neurons located in left and right DRG was considered as 100% (separately for left and right DRG).

During the present study, neuronal cells supplying the ICV were observed in bilateral DRG from neuromers Th7 to L4, and the differences between right and left DRG were not very clear (Figure 1).

The largest number of FB-positive cells was located in neuromers Th13 (16.88 ± 2.81% of all FB+ neurons) in the right DRG and 14.54 ± 3.07% in the left DRG). A slightly lower number of neuronal cells supplying the ICV was noted in neuromers Th9–Th12 (from 11.82 ± 2.90% to 12.41 ± 2.36% in right DRG and from 10.95 ± 2.26% to 13.16 ± 2.32% in left DRG) and L1–L2, respectively, 13.81 ± 2.58% and 9.70 ± 2.41% in right DRG, and 12.42 ± 3.85% and 10.37 ± 1.87% in left DRG). Significantly fewer neurons were present in neuromers Th7–Th8 (2.29 ± 2.10% and 5.18 ± 3.10% in right DRG and 2.34 ± 1.51% and 7.76 ± 1.81% in left DRG) and L3 (3.62 ± 1.71% in right DRG and 4.45 ± 2.51% in left DRG), and within the neuromer L4, FB+ neuronal cells were observed only sporadically (FB+ neurons were observed only in left DRG in quantities of 0.51 ± 1.15%). The total average number of FB+ neurons investigated in one animal amounted to 219.00 ± 67.30 (108.40 ± 35.98 in right DRG and 110.60 ± 32.24 in left DRG)

**Figure 2:**
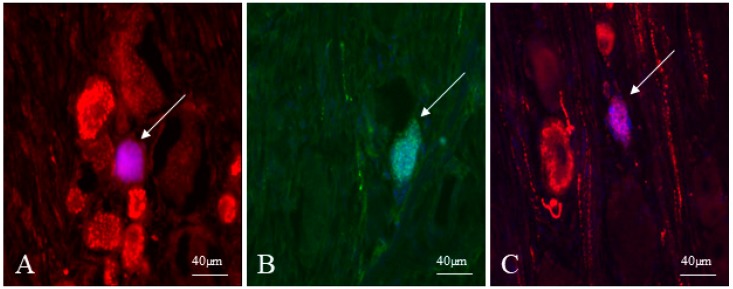
Fast blue-positive neurons in the dorsal root ganglia (DRG) supplying the ileocecal valve (ICV) (indicated with arrows) immunoreactive to calcitonin gene related peptide (CGRP) (**A**), substance P (SP) (**B**) and galanin (GAL) (**C**).

**Figure 3:**
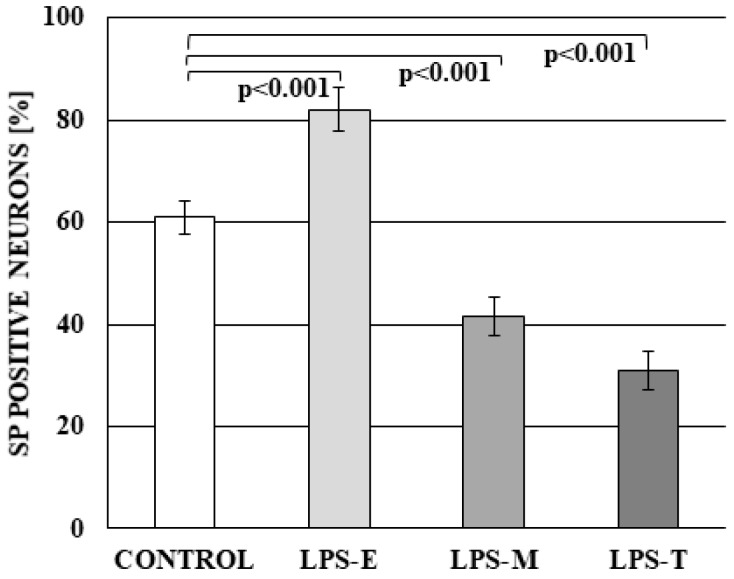
The percentage of neurons in dorsal root ganglia (DRG) immunoreactive to substance P (SP) in the control group and under the impact of LPS from *S.* Enteritidis (LPS-E), *S.* Minnesota (LPS-M), and *S.* Typhimurium (LPS-T). Statistically different at *p* < 0.001 as compared to the control group.

The highest number of sensory neurons supplying the ICV showed immunoreactivity to CGRP. The percentage of CGRP-positive cells amounted to 57.33 ± 6.89% of all FB+ cells (Figure 2A). The presence of SP was observed in a slightly lower percentage of the ICV projecting neurons (Figure 2B). This value reached 50.10 ± 8.71%. In turn, neurons immunoreactive to GAL were the least numerous. GAL was noted in 39.52 ± 5.33% of all FB-positive cells (Figure 3C).

Regarding in vitro culture, it should be underlined that we tried to count both all FB-labelled neurons projecting to the ICV and unlabelled neurons projecting to other parts of the intestine and other organs. Unfortunately, the number of FB-labelled neurons was too low to get reliable statistically results and, therefore, the results obtained from all the population of labelled and un-labelled neurons are presented in the present study.

Studies conducted on cell cultures have shown that LPS derived from all bacterial serotypes studied did not change the average number of neurons on a glass coverslip. The number of neurons in the control group amounted to 48.07 ± 7.62, whereas under the influence of LPS, they achieved 48.69 ± 7.85, 48.66 ± 8.92, and 48.10 ± 8.73 under the impact of LPS *S.* Enteritidis, *LPS S.* Minnesota and *LPS S.* Typhimurium, respectively.

Moreover, during the present study, the influence of LPS on neurons immunoreactive to SP and/or GAL was observed and changes clearly depended on bacterial serotype. In control animals, SP-positive cells amounted to 60.90 ± 3.34% of all neurons. LPS *S.* Enteritidis caused the increase of the number of SP + neurons to 82.09 ± 4.43%, whereas LPS *S.* Minnesota and LPS *S.* Typhimurium resulted in a decrease in the percentage of such cells (to 41.48 ± 3.67% and 30.83 ± 3.71%, respectively) (Figure 3). 

**Figure 4:**
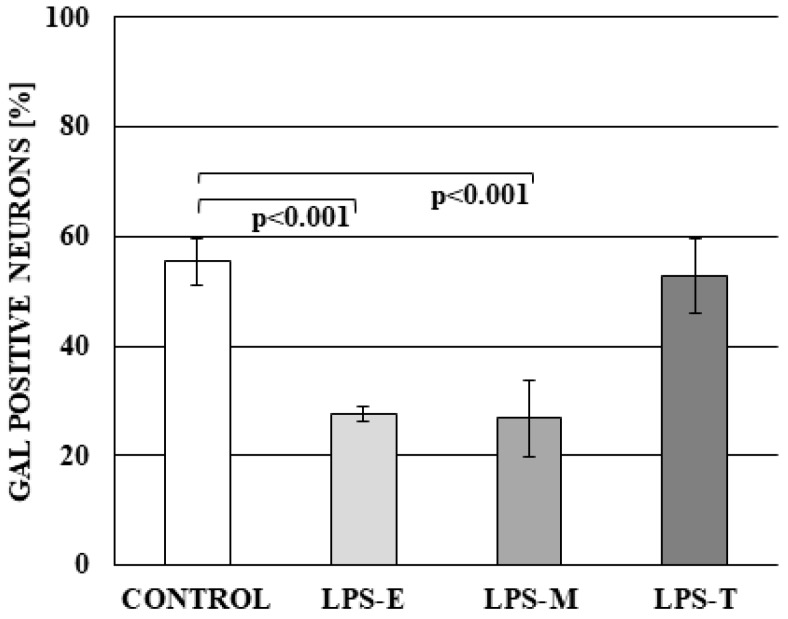
The percentage of neurons in dorsal root ganglia (DRG) immunoreactive to galanin (GAL) in control group and under the impact of LPS from S. Enteritidis (LPS-E), S. Minnesota (LPS-M), and *S.* Typhimurium (LPS-T). Statistically different at *p* < 0.001 as compared to the control group.

A different situation was observed in the case of GAL-positive neurons. In control animals, the percentage of such cells amounted to 55.44 ± 4.16%. LPS *S.* Enteritidis and LPS *S.* Minnesota caused the decrease in the number of GAL + neurons (to 27.51 ± 1.40% and 26.82 ± 7.08%, respectively), whereas under the impact of LPS *S.* Typhimurium, this value achieved 52.81 ± 6.80% and was not significantly statistically different from the percentage observed in the control group (Figure 4).

**Figure 5:**
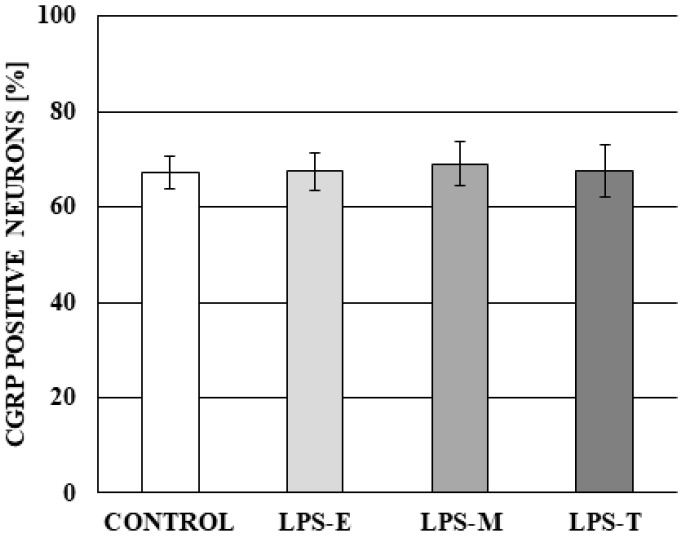
The percentage of neurons in dorsal root ganglia immunoreactive to calcitonin gene-related peptide (CGRP) in control group and under the impact of LPS from *S.* Enteritidis (LPS-E), S. Minnesota (LPS-M), and S. Typhimurium (LPS-T). Statistically significant differences for *p* < 0.001 as compared to the control group.

During the present investigation, the influence of LPS derived from all bacterial serotypes studied on the percentage of CGRP-positive neurons was not observed. The percentage of such neurons amounted to 67.19 ± 3.49% in control group, 67.52 ± 3.96% in LPS *S*. Enteritidis group, 69.08 ± 4.51% in LPS *S.* Minnesota group and 67.53 ± 5.35% in the LPS *S.* Typhimurium group. Differences between the mentioned above values were not statistically significant (Figure 5).

**Figure 6:**
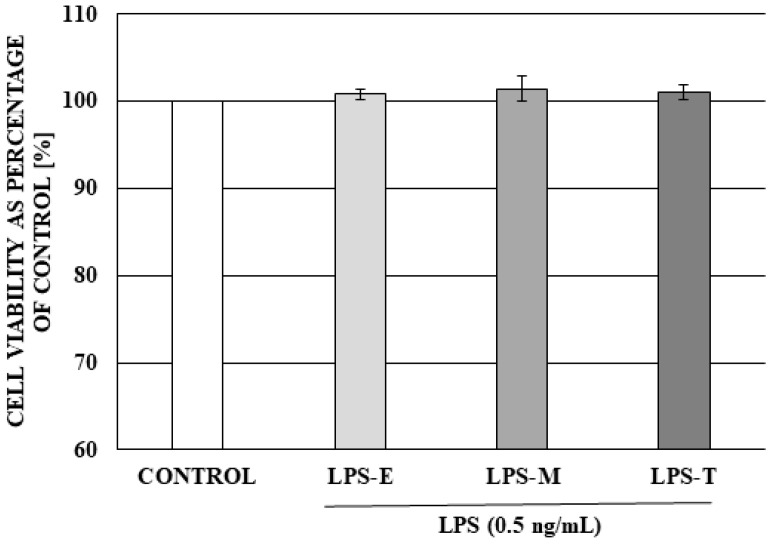
The effect of a low dose (0.5 ng/mL) of LPS from different serotypes of *Salmonella* spp: LPS from *Salmonella* Enteritidis (LPS-E), LPS from *Salmonella* Minnesota (LPS-M), LPS from *Salmonella* Typhimurium (LPS-T) on DRG cell viability. Cell viability was assessed using the MTT method. Data are presented as means of the percentage of the untreated control cells ± SE (*n* = 4).

The analysis of the cell viability (MTT assay) demonstrated that a 24 h exposure to the low dose of LPS from studied serotypes of *Salmonella* spp. did not exert any toxic or proliferative effect on neuronal and non-neuronal DRG cells viability of DRG (Figure 6).

**Figure 7:**
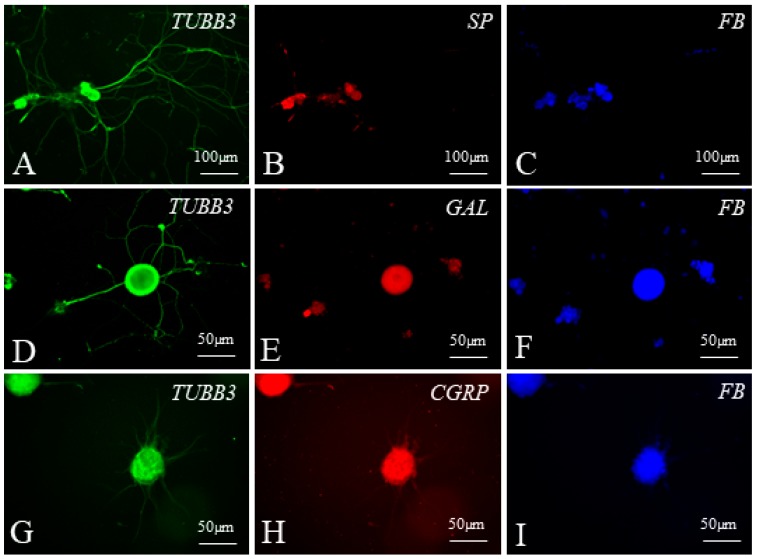
Examples of dorsal root ganglia (DRG) neurons supplying the ileocecal valve (FB-positive, blue) (**C**,**F**,**I**), immunoreactive to tubulin (TUBB3 used here as a neuronal marker, green) (**A**,**D**,**G**) and substance P (SP) (**B**), galanin (GAL) (**E**) or calcitonin gene-related peptide (CGRP) (**H**) in cell cultures of the control group.

**Figure 8:**
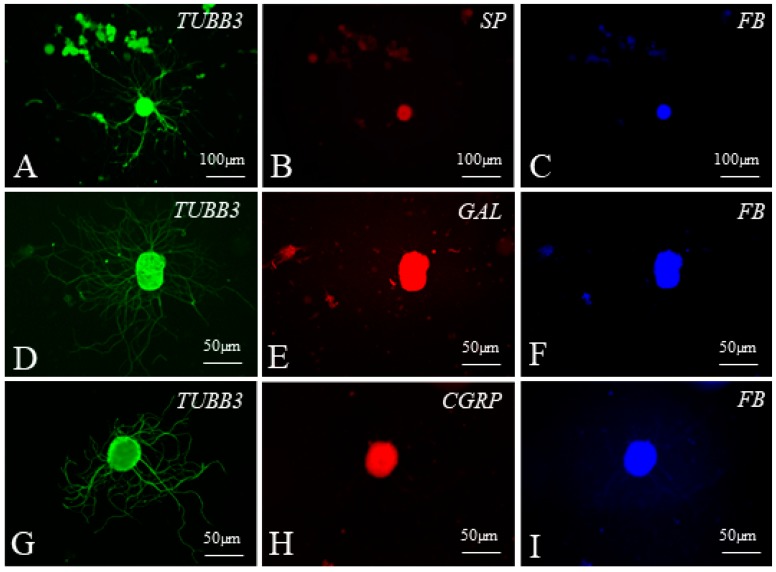
Examples of dorsal root ganglia (DRG) neurons supplying the ileocecal valve (FB-positive blue) (**C**,**F**,**I**) immunoreactive to tubulin (TUBB3 used here as a neuronal marker, green) (**A**,**D**,**G**) and substance P (SP) (**B**), galanin (GAL) (**E**), and calcitonin gene-related peptide (CGRP) (**H**) (all these substances in red) in cell cultures treated with lipopolysaccharides from *Salmonella* Enteritidis (**A**–**C**), *Salmonella* Typhimurium (**D**–**F**) and *Salmonella* Minnesota (**G**–**I**).

Representative examples of dorsal root ganglia (DRG) neurons supplying the ileocecal valve observed during the present study in the cell culture are visualised in Figure 7 and Figure 8. 

## 3. Discussion

The results obtained in this study indicate that the ICV, similar to other parts of the gastrointestinal tract, is supplied by neurons located in DRG [30]. Similar to previous studies, where DRG neuronal cells supplying the stomach, duodenum, ileum, and colon were described in various mammal species [30,31,32,33], observations made during the present investigations show that DRG plays a role in ICV sensory innervation in the domestic pig. On the other hand, the relatively low number of FB+ neurons observed in this study suggest that sensory components of the vagal nerve may play more important functions in the innervation of the ICV. This is according to the previous studies, where it was found that the network of processes derived from DRG neurons in the colon is more extensive than in the stomach and jejunum [30,32]. This is connected with the fact that branches of the vagal nerve supply the GI tract from the oesophagus to the proximal colon [34]. The majority of FB+ neuronal cells innervating the ICV investigated in this study were located in the thoracic and lumbar DRG, which is generally consistent with the distribution of previously described DRG neuronal cells supplying the small intestine.

Previous studies have shown that neuronal cells located in the DRG may contain (apart from typical substances involved in the sensory and pain stimuli conductions, such as SP and CGRP) a wide range of active neuronal factors, including GAL, CART peptide, vasoactive intestinal polypeptide, nitric oxide, somatostatin, and many others [35,36]. During the present study, the majority of DRG neurons supplying the ICV also contain SP and/or CGRP. SP is a member of the tachykinin family. It is a substance participating in sensory and pain stimuli conduction and has been observed in sensory neuronal cells and fibres in the central and peripheral nervous system of numerous species, including human [37,38,39,40,41]. It should be underlined that SP, besides sensory functions, may also participate in other various regulatory processes within the GI tract. In particular, it is known that SP regulates the intestinal motility and secretory activity [37], blood flow within the intestine and mesentery [42], as well immunological processes [43].

The second important substance participating in sensory and pain conduction, which has been observed in sensory neurons supplying the ICV, is CGRP. This peptide, similarly to SP, has been described in various central and peripheral sensory nervous structures of a wide range of mammals [44,45]. Within the digestive tract of some species, CGRP is considered to be a marker of intrinsic primary afferent neurons, which belong to the enteric nervous system and are a component of short intramural reflexes taking place without the central nervous system [46]. CGRP within the GI tract is also involved in regulatory processes connected with blood flow, secretory activity, and the absorption of nutrients [47,48]. Moreover, this peptide takes part in the protection of the intestinal mucosal layer against injuries and regulates intestinal motility [49].

The third substance studied in this investigation—GAL—is a not classical factor taking part in sensory or pain stimuli conduction. Nevertheless, the presence of GAL has been described in sensory neuronal structures supplying various internal organs [50,51]. It is known that GAL takes part in the regulation of other neurotransmitters by acting on the ion channels within the membrane of neuronal cells [52,53]. Besides sensory conduction, GAL in the GI system regulates the intestinal motility and secretory activity and the character of processes regulated by GAL clearly depends on the animal species and the fragment of the intestine [54,55].

In the light of the previous investigations, it is known that all neuronal factors studied during this experiment participate in regulatory processes not only under physiological conditions, but are also involved in pathological mechanisms of various diseases and intoxications with a wide range substances. Evidence of this includes changes in the expression of SP, CGRP, and GAL both in the enteric nervous system and extrinsic neuronal cells supplying the intestine under the influence of inflammatory processes, neuronal damage, intoxication with mycotoxins, and other toxicological substances and many other pathological stimuli [38,56]. For SP, these changes may be connected with neuroprotective properties and the participation of this peptide in inflammatory processes [38]. Namely, it is known that SP acting on lymphocytes and macrophages stimulates the secretion of pro-inflammatory cytokines, including IL-1 and TNF-α—the most important inflammatory mediator [37,43]. In turn, GAL is known from neuroprotective functions in the central and peripheral nervous system, which have primarily been described during brain injuries and neurodegenerative diseases [57]. Moreover, GAL (contrary to SP) plays an anti-inflammatory role by enhancing synthesis and secretion of IFN-γ and IL-12/23 while simultaneously decreasing the levels of TNF-α and IL-1β [58]. However, CGRP, whose role in intestinal diseases has not been fully elucidated, may inhibit the expression of TNF-α and IL-1β and take part in mechanisms connected with the development of diarrhoea [59]. On the other hand, the participation of CGRP in neuroprotective processes within the innervation of the intestine, despite changes in CGRP expression under various pathological stimuli [60], has not yet been confirmed.

Changes in the immunoreactivity of DRG neurons observed during the present study probably result from the above-mentioned functions of neuronal factors under the impact of LPS. These changes may be connected with neuroprotective and/or adaptive processes used to maintain homeostasis within the nervous tissues These may result from the pro-inflammatory activity of LPS, described in the previous investigations. In particular, it is known that one of the components of LPS —lipid A—strongly induces synthesis and the secretion of pro-inflammatory substances [61]. However, a more likely cause of the observed changes is the direct impact of LPS on neuronal cells. It should be underlined that this impact in the light of the previous studies is not clear and depends on LPS dose. On the one hand, the neurotoxic activity of LPS is relatively well-known. Previous investigations have described the involvement of this substance in neurodegenerative processes in various parts of the nervous system [62,63,64]. Moreover, LPS is used as a factor inducing experimental Parkinson’s disease in rodents [65,66,67,68]. On the other hand, it has been shown that low doses of LPS are essential to promote the survival of enteric and hippocampal neurons. Anitaha et al. [69] found that for enteric neuronal survival, microbial-neuronal interaction is essential and a low dose of LPS is essential to maintain neuronal survival, although at higher doses LPS results in neuronal toxicity. Low dose LPS treatment (10 ng/mL) promoted enteric neuronal survival through the activation of NF-κB and TLR4. None of our studied serotypes of *Salmonella* spp. influenced the neuron counts and furthermore, we saw that neurons treated with 0.5 μg/mL LPS have longer and denser neurites compared to the control group. This is only a subjective observation which has not been supported by any analysis of neuron morphology. Perhaps, hypothetically, it was in connection with the role of glial cells, but we did not study it because it was not the intended goal of our study. Additionally, LPS in a low dose has been shown to promote the survival of hippocampal neurons through increased expression of a granulocyte colony-stimulating factor [70]. But, in the central nervous system (CNS), hippocampal neurogenesis and neurological functions were attenuated by lipopolysaccharide-induced TLR4 activation [71]. Moreover, some studies have shown the protective effect of LPS pre-treatment [72] and endotoxin tolerance provides (enhances) the ischemic resistance of neuronal cells [73]. In contrast, Chen et al. [74] reported that pre-conditioning with a super-low or low dose of LPS exacerbates sepsis mortality. Further studies may be required to find consistent issues to combine the different results of various studies.

## 4. Materials and Methods

The present study consisted of two experiments conducted on nine immature sows of the Pietrain × Duroc breed. All animals were kept in standard laboratory conditions and fed with complete feeding stuff appropriate to the age and species of animals for two weeks prior to the experiment in order to allow adaptation to the new environment. The experiments took place when the pigs were 8–9 weeks of age with body weights of 16–18 kg. Both administration of the drugs and the performance of surgical procedures were performed by a veterinary doctor (DVM, Ph.D.). All procedures in the experiment were approved by the Local Ethical Commission of Experiments on Animals in Olsztyn (decision number 73/2015 from 29 September 2015).

### 4.1. Experiment No. 1: Localization and Neurochemical Characterization of Neurons Located in the Dorsal Root Ganglia and Supplying the ICV

The first experiment was conducted to study the sensory neurons located in dorsal root ganglia and supplying the ileocecal valve (ICV). For this part of the experiment, five pigs were used.

#### 4.1.1. Surgical Procedures Surgery

Before surgical procedures, five animals were pre-medicated using the method previously described by Mikolajczyk [75] with intramuscular injection of atropine (Atropinum Sulfuricum Polfa Warszawa S.A., Warszawa, Poland, 0.035 mg/kg b.w.), ketamine (Bioketan, Vetoquinol Biowet Sp. z o.o., Poland and Vetoquinol S.A., Lure, France, 7.0 mg/kg b.w.), and medetomidine (Cepetor, CP-Pharma Handelsges mbH, Burgdorf, Germany, 0.063 mg/kg b.w.). After 15 min. the animals were subjected to the general anaesthesia with propofol (Scanofol, NORBROOK, Newry, Northern Ireland, IRL.PN, 4.5 mg/kg b.w. given intravenously) and median laparotomy. During the transaction surgery procedure, a conventional midline incision of the abdominal wall was made. The cecum and ileum were identified and the ICV was isolated from the abdominal cavity. The ICV was injected with 50 μL of a 5% aqueous solution of the fluorescence retrograde neuronal tracer fast blue (FB; EMSChemie GmbH, Groß-Umstadt, Germany, ten injections, 5 μL each) using a Hamilton syringe equipped with a 26-gauge needle. Close attention was paid to avoiding any contamination of the surrounding tissues with FB due to the hydrostatic leakage from the injection canal. To avoid leakage, the needle was left in each site of FB injection for up to a minute. The peritoneum with the transverse abdominal muscles, the internal and external abdominal oblique muscles, and the cutaneous muscle with subcutaneous fascia were closed in a simple continuous pattern. The skin was closed in a subcuticular pattern.

#### 4.1.2. Sample Collection and Processing

After three weeks, the animals were again pre-medicated (as described above) and euthanized with pentobarbital (Morbital, Biowet Puławy Sp. z o.o, Poland, 60–70 mg/kg b.w., given intravenously). After death, the thoracic, lumbar, and sacral dorsal root ganglia were collected. Ganglia were fixed in 4% buffered paraformaldehyde (pH 7.4) for 30 min. and rinsed in phosphate buffer for three days (at 40 °C). They then added a 18% sucrose solution and stored it for least three weeks at 40 °C. After this period, the ganglia were frozen at −20 °C and cut into 10 μm-thick sections using a cryostat (HM 525, Microm International, Germany).

#### 4.1.3. Immunofluorescence Procedures with Counting Neurons

The sections were subjected to examination for the presence of neurons containing FB using a fluorescence Olympus BX51 microscope equipped with an appropriate filter set. Sections with FB-positive cells were subjected to typical single immunofluorescence technique by the method described previously by Gonkowski et al. [76]. Basically, during this method, fragments of DRG were subjected to (1) drying at room temperature (rt) for 1 h; (2) “blocking” in the solution containing 10% normal goat serum, 0.1% bovine serum albumin, 0.01% NaN3, Triton X-100 and thimerozal in PBS for 1 h (rt); (3) incubation with antibodies directed towards SP, CGRP or galanin (GAL) (overnight; rt, in a humid chamber); (4) incubation with secondary antibodies conjugated with appropriate fluorochromes (alexa fluor 594 or 488) to visualise the complexes “antigen—primary antibody”. The specifications and working dilution of primary and secondary antisera are presented in Table 1.

To confirm the specificity of the method routine standard controls, such as pre-absorption of the neuropeptide antisera with appropriate antigen, omission and replacement of primary antisera by non-immune sera were performed. To determine the percentage of neurons supplying the ICV immunoreactive to SP, CGRP, or GAL, at least 50 FB+ neuronal cells from each animal were evaluated for the presence of the particular neuronal factors. This relatively low number of neurons included in the experiment was caused by the relatively small number of all neuronal cells supplying the ICV. The obtained data was pooled, expressed as means ± SD.

### 4.2. Experiment No. 2: Culturing of Primary Sensory Neurons and Various Serotypes of Salmonella spp.

The second experiment conducted during this investigation consisted of a study of the influence of a low dose (0.5 ng/mL [77,78] of LPS from various types of *Salmonella* spp. serotypes: LPS from *Salmonella enterica* subsp. *enterica* serotype Enteritidis (L7770 Sigma), LPS from *Salmonella enterica* subsp. *enterica* serotype Minnesota (L4641 Sigma), LPS from *Salmonella enterica* subsp. *enterica* serotype Typhimurium (L6143 Sigma) on the neurochemical characterization of neurons located in DRG. Thus, during the in vitro experiment, four groups of cultures were used: control, Enteritidis (LPS-E), Minnesota (LPS-M), and Typhimurium (LPS-T). For this part of the experiment, four pigs were used.

#### 4.2.1. Sample Collection and Processing

After three weeks, all animals were again pre-medicated and subjected to general anaesthesia conducted as described above. During anaesthesia, the left and right thoracic (Th7–Th13) ganglia and lumbar (L1–L4) ganglia were exposed. For DRG exposure, an incision was made in the skin of the dorsal midline. The superficial muscular fascia was incised and the paraspinal muscles separated by a combination of sharp and blunt dissection, exposing the lumbar and thoracic vertebrae. A rongeur was used to remove bone fragments of spinal nerves as well as the intervertebral foramina from which they emerge, which were exposed along with particular ganglia. The animals were then euthanized with pentobarbital (in the above-described manner).

DRG were removed and transferred into cold RPMI 1640 W/HEPES W/GLUTAMAX-I medium (cat. no. 72400021, Life Technologies Polska Sp. z.o.o.; Warszawa, Poland) and antibiotic and antimycotic (cat. no. 15240062, Life Technologies Polska Sp. z.o.o.; Poland).

#### 4.2.2. Cell Culture and Treatments

Cell cultures were prepared as described previously [79,80]. Briefly, following removal of connective tissue, DRG were incubated in collagenase (cat. no. 17100017, Life Technologies Polska Sp. z.o.o.; Poland) for 60 min followed by 0.5% trypsin/EDTA (15400054, Life Technologies Polska Sp. z.o.o.; Poland). Neurons were dissociated by passages through a fire-polished Pasteur pipette and centrifuged at a low speed (10 min, 700 rpm). After final centrifugation, the pellet was suspended with TNB 100TM medium (cat. no. F8023; Biochrom AG, Berlin, Germany) containing: antibiotic and antimycotic and protein-lipid complex TM (cat. no. F8820; Biochrom AG, Germany). Equal volumes (50 µL) containing DRG neurons were seeded on glass coverslip (12 mm in diameter) coated with poly-D-lysine (0.01% solution; cat. no. P7280; Aldrich)/laminin (1 mg/mL L-2020, Sigma) placed in six-well multi-dishes (cat. no. 353046; BD Biosciences; Franklin Lakes, NJ, USA) at a density of 50–70 per glass. Cultures were cultivated in TNB medium 1 mL per well with 5 ng/mL nerve growth factor beta (NGF; N1408; Sigma Aldrich; Berlin, Germany) to maintain the survival of neurons [81]. After 36 h incubation at 37 °C in 5% CO_2_, the medium was replaced with fresh medium in the control and with fresh medium with the addition of 0.5 ng/mL LPS from *S.* Enteritidis, LPS from *S.* Minnesota (L4641 Sigma), LPS from *S.* Typhimurium (L6143 Sigma) in the LPS-treated group. We decided to use a 36 h cell primary neuron culture because extensive neurite outgrowth was observed after 24 h of culture [82]. 

#### 4.2.3. Immunocytochemical Labelling

After 60 h (including 24 h of culture with the addition of LPS to the media) in culture, neurons were fixed with 4% paraformaldehyde for 20 min, permeabilised with 0.01% Triton X-100 (X100-100ML; Sigma Aldrich; Germany) in PBS (P5493; Sigma Aldrich; Germany) for 5 min and blocked with blocking buffer (10% goat serum in PBS) for 30 min. DRG neurons were incubated with primary antibodies against substance P (8450-0004; AbD Serotec; Regensburg, Germany; 1:3000), calcitonin gene-related peptide (AB43873; Abcam; Cambridge, UK; 1:18,000), galanin (AB5909; Merck Millipore; Burlington, MA, USA; 1:16,400) and neuron-specific β-III tubulin (MAB1195; R&D Systems; Minneapolis, MN, USA; 1:1000) diluted in blocking buffer for 1 h at room temperature and then incubated with secondary Alexa-555- and Alexa-488-conjugated antibody (A31572; A-11001, Invitrogen, Carlsbad, CA, USA; 1:1000) for 60 min at RT. We stained all DRG neurons (FB-labelled and FB-unlabelled) using neuron-specific β-III tubulin [83] in co-localization with CGRP, SP, and GAL [84].

#### 4.2.4. Counting Cultured Neurons

Both FB-labelled neurons projecting to the ICV and unlabelled neurons projecting to other organs (also large extent, the colon) [85] were counted.

The experiment in vitro was performed in duplicate with two replicate wells (four coverslips in one well, i.e., eight coverslips per one animal) for each group of cultures (control, Enteritidis, Minnesota and Typhimurium). The number of neurons in the control and after LPS treatment group were counted and then expressed in percentage. FB-traced neurons were identified in the cell cultures by their blue fluorescence under the UV illumination using fluorescence Olympus BX51 microscope (V1 module, excitation range 330–385 nm and barrier filter at 420 nm). The number of β-III tubulin neurons in all studied groups (control and with the addition of LPS to the medium) was considered as the number of total neurons (100%). The number of neurons containing studied substances (β-III tubulin, CGRP, GAL, and SP) in intact and LPS-treated group was quantified using a fluorescent Olympus BX51 microscope (Shinjuku, Tokyo, Japan) equipped with an appropriate filter sets for Alexa 488 (B1 module, excitation filter 450–480 nm) and Alexa 555 (G1, excitation filter 510–550 nm). Microphotographs were acquired using 20× objectives and a PC equipped with a CCD camera operated by Cell Sens Dimension image analysis software (Olympus, Warsaw, Poland).

#### 4.2.5. MTT Assay

To determine the cell viability by MTT assay, DRG cells were prepared as described before using six well multi-dishes with glass coverslips and seeded in a 96-well plate (CytoOne cat no: CC7682-7596). After 36 h incubation at 37 °C in 5% CO_2_ the cells were treated with 0.5 ng/mL LPS from *S.* Enteritidis, LPS from *S.* Minnesota, and LPS from *S*. Typhimurium or tested without LPS (control group) and incubated at 37 °C in 5% CO_2_ for 24 h. After incubation, 50 μL MTT (Thiazolyl Blue Tetrazolium Bromide 5 mg/mL, M5655 Sigma) was added and the plates were incubated at 37 °C in 5% CO_2_ for 4 h. At the end of the incubation period, the supernatants were removed, and 100 μL of dimethyl sulfoxide (DMSO 34943, Sigma) was added to each well to enable the release of the blue reaction product-formazan. The absorbance at 570 nm was read on a microplate reader Infinite 200 (Tecan) and the results were expressed as a percentage of the absorbance measured in control cells and in the LPS-treated culture.

### 4.3. Statistical Analysis

All data were expressed as the mean ± standard deviation (SD) from independent experiments performed using five animals in the experiment 1 (*n* = 5) and four animals in experiment 2 (*n* = 4). Statistical analysis was determined using one-way analysis of variance (ANOVA) followed by Tukey’s test for multiple comparisons (Statistica software /version 13.1 /StatSoft, Cracow, Poland). 𝑃 values less than 0.05 were considered significant.

## 5. Conclusions

To sum up, the results obtained during the present study have shown that sensory processes supplying the ICV may derive from neurons located in DRG from neuromers Th7 to L4. The ICV sensory neurons contained SP, CGRP, and GAL. Moreover, it has been shown that the dose of LPS used in the in vitro study did not change the number of DRG neurons from neuromers Th7 to L4 supplying the ICV and other organs and tissues, but influenced their neurochemical characterization. The observed changes clearly depended on the bacterial serotype of LPS. This is proof that structural differences of LPS—not only between bacterial species, but also within particular serotypes of one species—result in varied impact on the nervous system. Moreover, the obtained results have shown that SP and GAL, contrary to CGRP, are involved in the processes connected with the impact of LPS. Changes in the expression of these substances may result from the direct influence of LPS on the nervous structures and/or pro-inflammatory activity of LPS. However, the explanation of the exact mechanisms connected with LPS-induced fluctuations in neurochemical characterization of DRG neurons requires further study.

## Figures and Tables

**Table 1:** List of antisera and reagents used in immunohistochemical investigations.

**Primary antibodies**				
Antigen	Code	Species	Working dilution	Supplier
CGRP	AB5920	Rabbit	1:1600	Chemicon Int Temecula, OH, USA
GAL	T-5036	Guinea Pig	1:2000	Peninsula San Carlos, CA, USA
SP	8450-0505	Rat	1:1000	Bio-Rad (AbD Serotec), Kidlington, UK
**Secondary antibodies**				
Reagents			Working dilution	Supplier
Alexa fluor 546 donkey anti-rabbit IgG			1:1000	Invitrogen Carlsbad, CA, USA
Alexa fluor 546 donkey anti-guinea pig IgG			1:1000	Invitrogen
Alexa fluor 488 goat anti-rat IgG			1:1000	Invitrogen
